# Available sustainable alternatives replace endangered animal horn based on their proteomic analysis and bio-effect evaluation

**DOI:** 10.1038/srep36027

**Published:** 2016-10-27

**Authors:** Rui Liu, Fei Wang, Qiong Huang, Jin-ao Duan, Pei Liu, Erxin Shang, Dong Zhu, Hongmei Wen, Dawei Qian

**Affiliations:** 1 Jiangsu Collaborative Innovation Center of Chinese Medicinal Resources Industrialization, and National and Local Collaborative Engineering Center of Chinese Medicinal Resources Industrialization and Formulae Innovative Medicine, Nanjing 210023, P.R. China; 2 Jiangsu Key Laboratory of Research and Development in Marine Bio-resource Pharmaceutics, Nanjing University of Chinese Medicine, Nanjing 210023, P.R. China; 3 Jiangsu Key Laboratory for High Technology Research of TCM Formulae, Nanjing University of Chinese Medicine, Nanjing 210023, P.R. China; 4 School of Pharmacy, Jiangsu University, Zhenjiang 212013, P.R. China; 5 Suzhou Hospital of Traditional Chinese Medicine, Suzhou, 215009, P.R. China; 6 The First Affiliated Hospital, Nanjing Medical University, Nanjing 210029, P.R. China

## Abstract

The use of endangered animal products in traditional Chinese medicine (TCM) and other ethno-medicines is culturally widespread across many regions of Asia. In the present study, traditional efficacies of seven types of animal horn including antipyretic, sedative and procoagulant activities were evaluated. Shotgun proteomic analysis was performed on material from horns following separation into soluble and insoluble fractions. Over 200 proteins were identified in each sample using nano LC-MS/MS, and these were classified according to their molecular function and cellular component using principal component analysis (PCA). The results indicated that seven horns showed antipyretic, sedative and procoagulant effect. Proteomic analysis showed that YH and WBH were similar to RH in terms of protein profile, and GH was similar to SAH. In addition, YH and GH were similar to RH in their cellular component classification profile. PCA based on the composition of keratin and keratin-associated proteins showed that constituents of WBH and GH were similar to RH and SAH, respectively. This is the first analysis of the protein content of animal horns used in TCM, and it is effective to substitute the horn of endangered animals with sustainable alternatives from domestic animals.

The horns of rhinoceros (*Rhinoceri Asiatici Cornu*, RH), Saiga antelope (*Saigae Tataricae Cornu*, SAH) and water buffalo (*Bubali Cornu*, WBH) have been applied in traditional Chinese medicine (TCM) for over 2000 years^1^,^2^. However, with increasing human population and demand, illegal hunting has led to drastic reductions in rhinoceros and Saiga antelope populations in recent decades^3^. Even though five species of rhinoceros are listed as endangered according to the International Union for Conservation of Nature (IUCN) and Appendix I of the Convention on International Trade in Endangered Species (CITES)^4^,^5^, the law seems to have done little to save these species, and the African rhinoceros population has declined from 65,000 to less than 3500 over the last 40 years. Indeed, the rhinoceros population across the entire world decreased from 70,000 to 11,000 between 1970 and 1987, a dramatic drop of 85%^1^. Similarly, a place on Appendix II of the CITES list for the Saiga antelope failed to protect this species, and the population has fallen drastically from 1 million in the 1980s to only 25,000 in 2000. This species was placed on the endangered species list in 2001^3^,^4^.

The use of RH and SAH is widespread in TCM, and both are considered indispensable for various remedies and applications. It has been reported that aqueous extract of RH and SAH possess notable antipyretic properties^1^,^2^,^6^. However, the application of RH and SAH is illegal or restricted since 1970’s. Developing substitutes would resolve the conflict between legislation and the apparent indispensability of RH and SAH in TCM applications, and is important for protecting these highly endangered species^7^. In order to change the culture of using materials derived from endangered animals in TCM, increasing numbers of scientists have begun investigating the potential use of horns from non-endangered, abundant and domesticated species as replacements for RH and SAH.

There are scientists proposed to search for substitutes of threatened animal organs based on their pharmacological testing^8^,^9^. In view of the pharmacological studies and clinical applications, animal horns such as WBH, goat horn (GH), yak horn (YH), etc. were widely used as substitutes of RH or SAH^10^,^11^,^12^. Luo reported that GH possesses similar *in vitro* antithrombotic and anticoagulation activities to SAH and is therefore a rational substitute^7^. But reported studies in rats that both WBH and cattle horn extracts showed significant antipyretic action at a dosage of 5 g/ml, and the traditionally prescribed *Qingying Decoction* also exhibited positive antipyretic activity when RH was replaced by WBH^1^,^2^. Another traditional prescription ‘*Xijiao Dihuang* decoction’ was documented in *Thousand Golden Prescriptions* (*Tang* dynasty, 581–682 A.D.), in which RH was the most important medicine. Currently, RH in *Xijiao Dihuang* decoction was normally replaced by WBH and showed similar clinical curative effect. It was also reported that both SAH and GH showed apparent sedative activity and antihypertensive activity^11^. In 1987, Ministry of Health of China issued a document concerned using GH, *Gazella subgutturosa* horn and *Mongolian gazelle* horn as medicinal materials (*Gazella subgutturosa* and *Mongolian gazelle* cannot be used because they are species under second rank state protection in China, thus, only GH can be used in clinic and pharmaceutical industry) due to their similar effects compared with SAH. In addition, in order to achieve considerable efficacy, the dosage of WBH and GH used as substitutes of RH and SAH should be 5–10 fold, or even more than 10 fold^1^,^2^,^11^.

Animal horns are pointed projections of the skin on the heads of animals consisting of a covering of horn sheath surrounding a core of living bone. Unlike the horns of Antelope, water buffalo and other Artiodactyla animals, RH lacks the bony core and is composed entirely of horn material^13^. Animal horn is structurally similar to mammalian hair and nail plate and avian epidermis, and is comprised of a complex mix of protein and peptides involving disulfides and isopeptide cross-links^14^,^15^. Keratins and keratin associated proteins (KAPs) constitute the bulk of the horn cortex^16^. In horn medulla and cuticle cells, transglutaminase forms extensive ε-(γ-glutamyl) lysine cross-links resulting in a highly insoluble material that is difficult to examine^17^.

Despite increasing research on WBH, GH, YH, etc. applied as alternatives of RH or SAH in TCM clinic, mechanisms of action remain unknown. The complex structure of animal horn makes it difficult to identify and separate the active components. Fortunately, the shotgun technique is known to be compatible with corneous materials such as hair, nail, and wool, and proved to be suitable for animal horn. Therefore, in the present study, shotgun proteomic analysis was applied to analyze constituents of animal horns. The large-scale identification of protein components is necessary for a molecular-level understanding of this complex material. In recent years, advances in mass spectrometry and database searching have assisted the identification of proteins in complex cross-linked structures^18^,^19^. In particular, shotgun proteomic analysis has been applied successfully for investigating human hair, nail plate, and chicken corneocytes.

Furthermore, according to the documentation of *Bencao Gangmu* (*Ming* dynasty, 1578 A.D.) or some other traditional literatures, animal horn derived TCMs were usually applied for treating *heat* syndrome, relieving convulsion, and reducing blood bleeding. Therefore, bio-effect evaluation of animal horns including antipyretic, sedative, and procoagulant activities were evaluated in the present study, which may help to illustrate the bio-effect characteristics of various animal horns.

## Results and Discussion

### Antipyretic activity test

**Figure 1:**
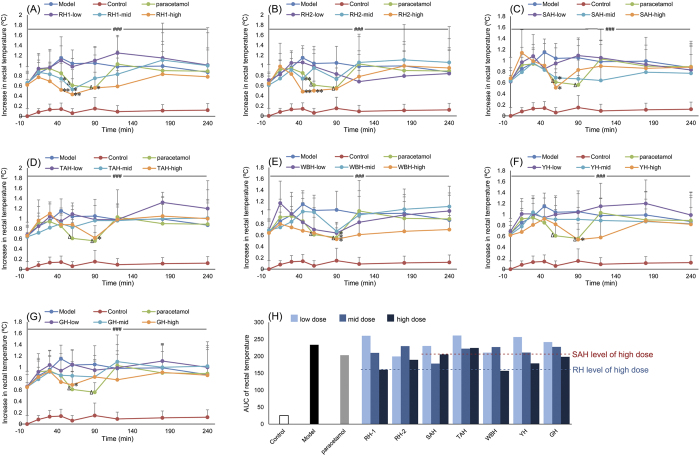
Effects of seven horns and paracetamol on endotoxin-induced fever rabbits. Values were expressed as mean ± SD (n = 6). (**A**–**G**) were rabbit temperature changing curves after administration horns, (**H**) was the AUC values of seven horns. ^###^*p* < 0.001, compared with Control group; *p < 0.05, **p < 0.01, compared with Model group; ^Δ^*p* < 0.05, Aspirin group compared with Model group.

The effect of animal horns on the fever rabbit was shown in Fig. 1 and Table S31. Compared with Model group, seven horns significantly reduced the temperature of fever rabbits after oral administration. RH-1 and RH-2 at high dosage exhibited strong antipyretic effect 45–90 min after oral administration (*p* < 0.05, *p* < 0.01). As shown in Fig. 1(H), the antipyretic efficacy strength was quantified by calculating the AUC of rectal temperature changing curve, and the AUC_240 min_ of each dose of animal horns was calculated. Among the seven horns, only the AUC_240 min_ of Tibetan antelope horn (TAH) at three doses was higher than paracetamol. The antipyretic efficacy strength depicted by AUC_240 min_ at high dose showed the order of seven horns was as follow: WBH > RH-1 > YH > RH-2 > GH > SAH > TAH. Based on these results, it is obvious that RH exhibit the strongest ability in lowering down rabbit body temperature. In addition, WBH continuously lower down the rabbit body temperature during the 240 min, whose AUC value was the lowest among seven horns at high dose level. In the present study, dosage of animal horns was identified according their clinical use, the high dosage of RH and SAH was 0.06 g/kg, while WBH and YH was 1.5 g/kg. Therefore, the dose of WBH or YH was 25 times higher than RH. At the present dose level, the AUC_240 min_ of WBH or YH was similar with RH. It is indicated that WBH or YH exhibit similar efficacy strength compared with RH in the antipyretic activity test. It is consistent with the fact of WBH clinical dose over 10-fold higher than RH. RH, WBH and YH also can be observed stronger antipyretic efficacy than SAH, TAH and GH.

**Figure 2:**
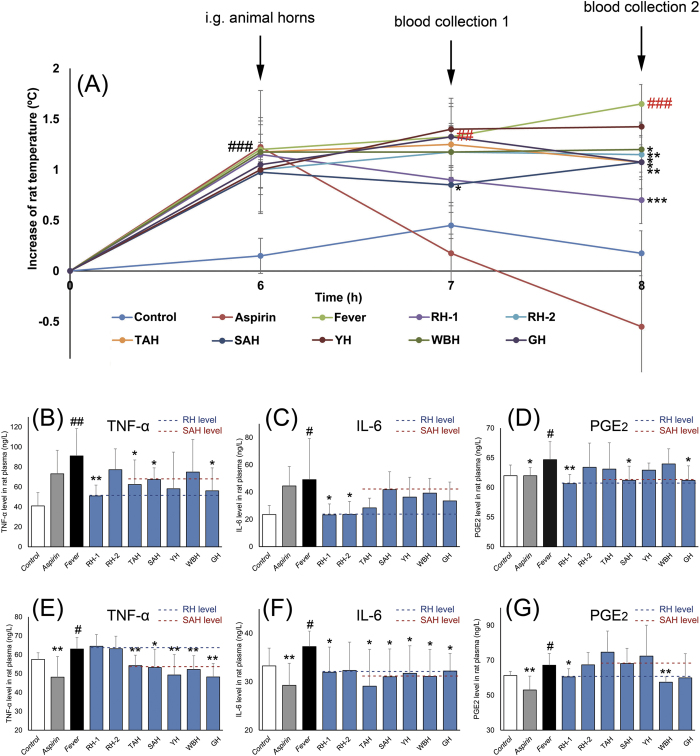
Effects of seven horns and Aspirin on yeast-induced fever rats. Values were expressed as mean ± SD (n = 8). (**A**) was rat temperature changing curves after administration horns, (**B**) TNF-α level in rat plasma 1 h after administration horns; (**C**) IL-6 level in rat plasma 1 h after administration horns; (**D**) PGE_2_ level in rat plasma 1 h after administration horns; (**E**) TNF-α level in rat plasma 2 h after administration horns; (**F**) IL-6 level in rat plasma 2 h after administration horns; (**G**) PGE_2_ level in rat plasma 2 h after administration horns.

The effect of animal horns on the fever rat was shown in Fig. 2 and Table S33. Compared with Model group, SAH could significantly reduce the temperature of fever rats 1 h after oral administration (*p* < 0.05). RH-1, RH-2, SAH, TAH, WBH and GH could significantly reduce the temperature of fever rats 2 h after oral administration (*p* < 0.05, *p* < 0.01, *p* < 0.001). Furthermore, TNF-α, IL-6, and PGE_2_ levels in the plasma of rats were determined. One hour after oral administration with seven horns, RH-1 could significantly reduce the levels of TNF-α, IL-6, and PGE_2_ (*p* < 0.05, *p* < 0.01), RH-2 could reduce IL-6 level (*p* < 0.05), SAH and GH could reduce TNF-α and PGE_2_ level (*p* < 0.05), TAH could reduce TNF-α level (*p* < 0.05). Two hours after the oral administration, RH-1 also showed effects on reducing IL-6, and PGE_2_ level (*p* < 0.05), SAH, TAH, WBH, YH and GH could significantly reduce the levels of TNF-α and IL-6 (*p* < 0.05, *p* < 0.01), in addition, WBH significantly reduced the PGE_2_ level (*p* < 0.05). Fever model induced by yeast is considered as an infectious fever model and the inflammatory cytokines, such as TNF-α, IL-1β, IL-6, PGE_2_, etc. were stimulated by yeast, and then cytokines elevate the body temperature. These cytokines play key roles in regulating fever. Therefore, TNF-α, IL-6, and PGE_2_ in the plasma of fever rats were determined after treating with animal horns in the present study. As shown in the results, RH-1 could reduce the levels of TNF-α, IL-6, and PGE_2_ in two periods after oral administration. Compared with RH-1, WBH and YH do not show significant cytokines-reducing efficacy in the first period after oral treatment. Two hour after WBH or YH oral treatment, levels of TNF-α, IL-6, and PGE_2_ decreased significantly. It can be indicated that RH-1, WBH, and YH can relieve fever by reducing levels of cytokines, only but RH-1, WBH and YH exert their antipyretic effect at different time after the treatment. Furthermore, SAH and GH showed similar cytokines-reducing efficacy. In two different periods after oral treatment with both SAH and GH, the levels of cytokines significantly decreased. As available sustainable alternatives, WBH, YH and GH could reduce the levels of cytokines in the plasma of fever rats, which showed similar cytokines-reducing efficacy compared with RH and SAH.

### Sedative activity test

**Figure 3:**
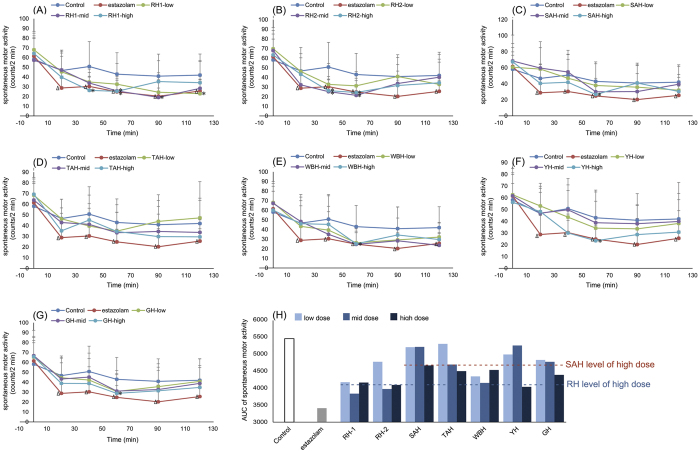
Effects of seven horns and estazolam given on spontaneous motor activity in mice. Values were expressed as mean ± SD. (n = 10). (**A**–**G**) were mice spontaneous movements changing curves after administration horns, (**H**) was the AUC values of seven horns. *p < 0.05, **p < 0.01, compared with Control group; ^Δ^*p* < 0.05, Estazolam group compared with Control group.

As shown in Fig. 3 and Table S32, oral administration with RH, SAH, WBH, YH and GH showed significant activity on decreasing mice voluntary movements (*p* < 0.05), TAH could decrease the mice spontaneous movements, but showed no significant difference with control (*p* > 0.05). 40 min after oral administration with horns, RH-1 and RH-2 significantly decreased mice voluntary movements (*p* < 0.05), 60 min after administration, RH, SAH, WBH, YH and GH could decreased mice voluntary movements significantly (*p* < 0.05). As shown in Fig. 3(H), the sedative efficacy strength was quantified by calculating the AUC of mice spontaneous movements changing curve, the AUC_120 min_ of each dose of animal horns was calculated. According to the AUC_120 min_ value at high dosage, the sedative efficacy strength order was as follow: YH > RH-2 > RH-1 > GH > TAH > WBH > SAH. The AUC value could be used to evaluate the continuous effects, not only an efficacy data at certain time point. As a result, YH exhibit better sedative effect than RH and SAH based on the high dose AUC_120 min_ value. AUC_120 min_ value of GH and TAH was higher than SAH, which indicated that GH or TAH showed better sedative effect than SAH, despite TAH did not significantly decrease the mice movements at each time point. As a result, in general, sedative effect of RH, WBH and YH was better than GH, TAH and SAH.

### Procoagulant activity test

**Figure 4:**
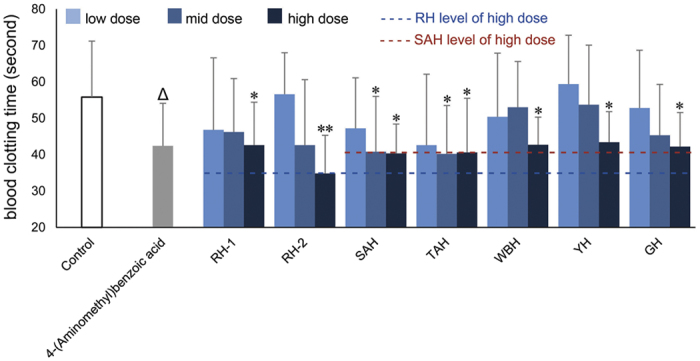
Procoagulant effect of seven horns and 4-(aminomethyl) benzoic acid. *p < 0.05, **p < 0.01, compared with Control group; ^Δ^*p* < 0.05, 4-(aminomethyl)benzoic acid group compared with Control group.

As shown in Fig. 4, oral administration with seven horns decreased blood clotting time significantly at high dose (*p* < 0.05; *p* < 0.01). According to the results, the order of procoagulant activity at high dose was as follow: RH-2 > SAH > TAH > GH > RH-1 > WBH > YH. Except RH-2, the procoagulant activity of other six horns was similar. However, at mid dose the order of procoagulant activity was described as: TAH > SAH > RH-2 > GH > RH-1 > WBH > YH. RH-2, SAH and TAH exhibit significant procoagulant activity and the procoagulant efficacy of WBH and YH was slightly lower than other horns.

Seven types of horn all possess antipyretic, sedative and procoagulant activity, which confirmed their documented traditional efficacies. All their dosage was identified according to the traditional literature, such as *Bencao Gangmu*, *Shennong Bencao Jing* (*Han* dynasty, about 200 B.C.–200 A.D.). Under their recommended dose level, horns all exhibit three traditional bio-effects above (*p* < 0.05; *p* < 0.01), but represent different efficacy priorities. RH-1 and RH-2 exhibit excellent antipyretic and sedative effect, followed by WBH and YH which exhibit satisfied antipyretic and sedative effect. At the high dosage level, RH, WBH, SAH, and GH could significant reduce the levels of cytokines, such as TNF-α, IL-6, and PGE_2_. The antipyretic and sedative effect WBH or YH was slightly lower than RH. Although SAH, TAH and GH also showed antipyretic and sedative effect, their effect was lower than RH, WBH or YH. In addition, RH-2, SAH and TAH exhibit apparent procoagulant activity, which slightly better than WBH and YH. Therefore, it was indicated that RH possesses wonderful antipyretic, sedative and procoagulant activity, WBH and YH tend to show significant antipyretic and sedative activity, while SAH, TAH and GH tend to exhibit significant procoagulant activity.

### Extraction of material from animal horns

**Figure 5:**
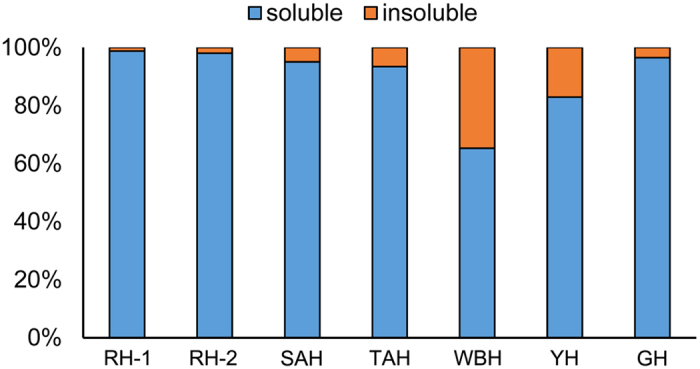
Proportion of soluble and insoluble material following extraction with SDS-DTT.

Animal horns such as RH, SAH, GH and WBH have been used in TCM for thousands of years, and TAH and YH have been used as ethnomedicines in Tibet for a comparably long time. In order to investigate the protein content and provide evidence for finding suitable sustainable substitutes to these endangered animal parts, we analyzed the proteomes of seven animal horns (RH-1, RH-2, SAH, GH, WBH, TAH and YH). In TCM, animal horns are usually powdered and swallowed with water directly, or extracted and decocted for oral administration^6^,^20^. Following oral administration, soluble components can therefore dissolve and exert their physiological effects. In the present study, samples were extracted exhaustively with SDS-DTT under reducing conditions to separate soluble and insoluble fractions. The soluble fraction of RH accounted for 98%, which was the highest soluble content of all seven animal horns studied, while only 65% of the WBH sample was soluble, which was the lowest (Fig. 5).

### Protein identification

**Figure 6:**
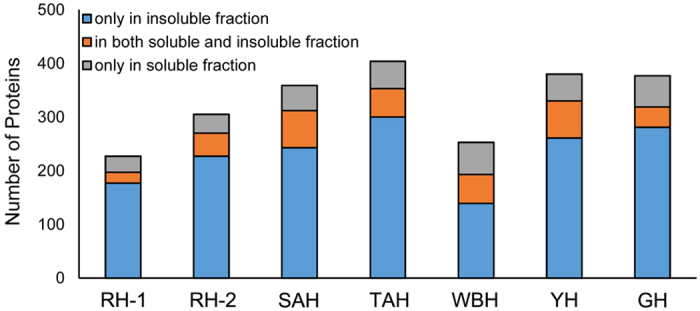
Number of proteins present in soluble and insoluble fractions.

Among the proteins identified, keratins and KAPs, junctional proteins, ribosomal proteins, initiation/elongation factors, histones, heat shock proteins and 14-3-3 proteins were abundant. Between 12.6% and 23.7% of these proteins were present only in the solubilized fraction, while 54.9%–78.0% were only in the particulate fraction, and 8.8%–21.3% were observed in both fractions (Fig. 6). The highest number of proteins were identified in TAH (404), and RH-1 produced the fewest (227). Keratin and KAPs were detected in both the soluble and insoluble fraction in all horn samples. Type I and II keratins of cuticular origin were apparent, along with other keratins (Table S1).

Desmosomal structural proteins such as desmoplakin, plakophilin, desmoglein, desmocollin, envoplakin and plakoglobin were identified, all of which play important roles in structural aspects of the cytoskeleton or in providing essential adhesion structures and linking transmembrane desmosomal cadherins to the cytoplasmic keratin filament network^21^. Other structural proteins including histones, tubulin, actin and catenin were also detected, and heat shock proteins (HSP) that function in cellular stability were also apparent. Both the 60S 40S ribosomal proteins that are crucial for protein synthesis were identified, along with elongation factor and eukaryotic translation elongation factor that regulate translation. Enzymes identified include pyruvate kinase, creatine kinase, and phosphoglycerate kinase, and numerous proteins involved in binding activities were detected based on their GO categories, such as pyruvate kinase binding with metal ion, tubulin binding with nucleotide, and junction plakoglobin binding with proteins.

GO analysis indicated that many of the identified proteins in the solubilized fraction (17.6%–24.8%) were related to the cytoskeleton, including keratins and KAPs that were the most abundant structural proteins in animal horns, and junction plakoglobin and tubulin. Keratins form a cytoplasmic network of intermediate filaments and KAPs interact with these filaments and stabilize disulfide linkages.

The insoluble fractions contained between 23.8% and 28.5% cytoplasmic proteins including type I cytoskeletal keratin, plakoglobin, elongation factor, tubulin, and 40S and 60S ribosomal proteins. These results suggest that most keratins are solubilized, however the high degree of cross-linking results in a large number of soluble proteins interacting with or co-localizing with the insoluble material, and this may be functionally important in horn epidermal cells^14^.

### Functional classification of animal horn proteome profiles

**Figure 7:**
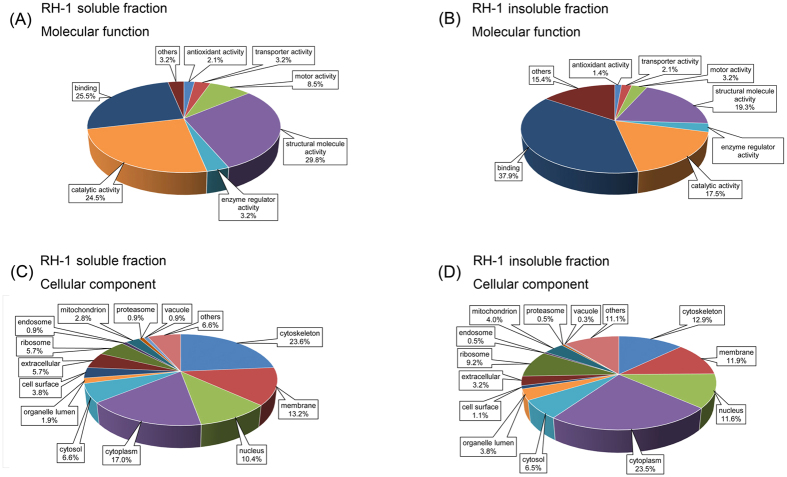
Gene Ontology analysis of identified proteins of *Rhinoceri Asiatici Cornu*. Protein sets (**A**,**B**) were classified according to molecular function, while (**C**,**D**) were classified according to cellular component.

In the case of RH, 227 proteins were identified, of which 60 were found exclusive to the soluble fraction, and 208 proteins were detected only in the insoluble fraction. Among the identified proteins in the soluble fraction, over 95% were assigned to a predicted function and classified into seven categories according to molecular function. The majority of these (28, 29.5%) were classified as structural proteins, and 21 of these were keratins (Fig. 7). Other abundant groups included those with binding activity (25 proteins, 25.3%) and catalytic activity (23, 24.2%). The remaining proteins possessed motor activity (eight, 8.4%), transporter activity (three, 3.2%), enzyme regulator activity (three, 3.2%) and antioxidant activity (two, 2.1%). The molecular function of proteins identified in the other six animal horns was similarly assigned (Tables S2–S29).

Of all proteins identified in the insoluble fraction, approximately 85% were assigned to various molecular functions. The majority of these were classified as having binding activity (108 proteins, 37.9%) including protein binding, nucleotide binding, metal ion binding, and DNA or RNA binding (Fig. 7). The functions of others included structural activity (55 proteins, 19.3%), catalytic activity (50 proteins, 17.5%), enzyme regulator activity (nine proteins, 3.2%), motor activity (nine proteins, 3.2%), transporter activity (six proteins, 2.1%), and antioxidant activity (four proteins, 1.4%).

Cellular component classification analysis of the 227 identified RH proteins placed 60 soluble fraction proteins into 12 categories (Fig. 7). The majority of these proteins were located in the cytoskeleton (25 proteins, 24.0%), cytoplasm (18, 17.3%), membrane (14, 13.5%) and nucleus (11, 10.6%). Others were located in the cytosol (seven, 6.7%), extracellular (six, 5.8%), ribosome (six, 5.8%), cell surface (four, 3.8%), mitochondria (three, 2.9%), organelle lumen (two, 1.9%), endosome (one, 1.0%) and vacuole (one, 1.0%). The 208 proteins identified in the insoluble fraction were classified as follows: the majority were localized to the cytoplasm (87 proteins, 23.8%), cytoskeleton (48, 13.2%), membrane (44, 12.1%) and nucleus (43, 11.8%). Others were localized to ribosomes (34, 9.3%), cytosol (24, 6.6%), mitochondria (15, 4.1%), and 12 (3.3%) were extracellular.

The proportion of soluble proteins with a structural function was higher than the insoluble fraction. The soluble fraction mainly consisted of keratins, while many of the insoluble proteins belonged to the binding activity category. The other animal horn samples were subjected to the same analysis, and the similarities and differences between the seven animal horn samples were investigated using PCA.

**Figure 8:**
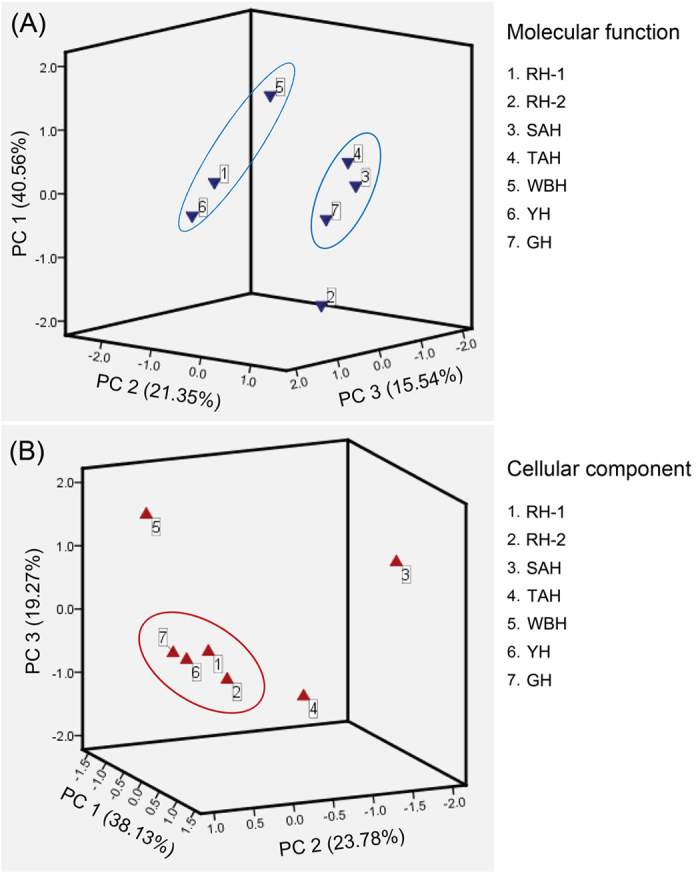
PCA based on molecular function and cellular component classification profiles.

PCA of molecular function reduced eight categories into three principal components (PC 1, PC 2 and PC 3) which accounted for >77% of the entire variance (Fig. 8A). Based on molecular function (Supplemental data), RH-1, YH and WBH were classified into one group, and SAH, TAH and GH were classified into another group. This suggests that YH and WBH may be similar to RH-1 at the level of protein molecular function, while GH may be similar to SAH. Furthermore, PCA of the cellular component reduced 13 categories into three principal components (PC 1, PC 2 and PC 3) which accounted for >81% of the variance (Fig. 8B). RH-1, RH-2, YH and GH were classified into one group, indicating similarities between YH and GH with RH in terms of their cellular component classification profile.

**Figure 9:**
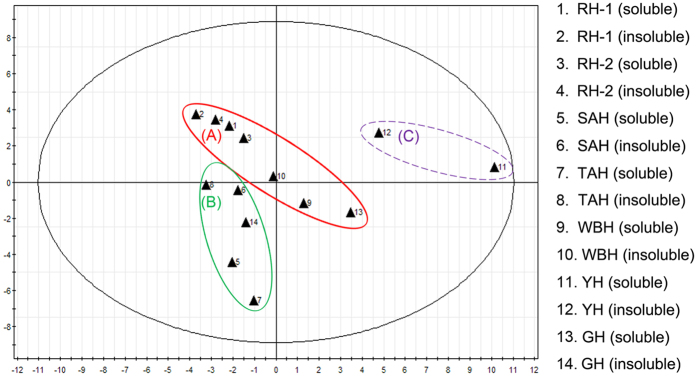
PCA based on the composition of keratin and KAPs in soluble and insoluble fractions of animal horns.

The PCA model generated by Simca-P was used to investigate sample classification, and the *R2X* of the PCA model was 0.771. The score plots of the PCA model (Fig. 9) revealed a distinct clustering among all soluble and insoluble fractions, which were separated into three regions based on the number of keratin and KAP peptides. Based on the results of PCA, both soluble and insoluble fractions of RH-1, RH-2 and WBH, and the soluble fraction of GH were classified in group A; both soluble and insoluble fractions of SAH and TAH, and the insoluble fraction GH were classified in group B; YH was classified alone in group C. PCA of keratins and KAPs demonstrated a high degree of similarity among all horn samples. WBH and GH were more closely related to RH-1 and RH-2 than YH, and GH was more similar to SAH and TAH than YH and WBH. These results suggested that the keratin constituents of WBH and GH were similar to RH and SAH, respectively. It is therefore why WBH was used as substitute of RH, and GH was applied for replacing SAH in clinical trials and TCM.

### Available sustainable alternatives of endangered animal horns

It was first documented in *Shennong Bencao Jing* that RH and SAH possess potent antipyretic and detoxification activities^1^,^2^,^22^. These substances have been used in TCM for over 2000 years and are believed to be indispensable. However, both the rhinoceros and Saiga antelope have experienced massive population crashes and are now threatened with extinction, and using parts from these animals is now illegal^4^. Researchers have begun to investigate horns from other animals as possible substitutes for RH and SAH^7^,^8^,^9^,^12^,^23^,^24^. As a result, bio-effects evaluation showed that seven horns in the present study all possess apparent antipyretic, sedative and procoagulant activity. However it should be remembered that although it may be possible to replace one activity of RH or SAH, it might not be possible to replace all of the various bioactivities. For instance, the antipyretic and sedative effect of WBH and YH showed slight better than SAH, TAH and GH, while procoagulant activity of SAH, TAH and GH was higher than WBH and YH. Therefore, it might be available to substitute RH with WBH or YH in treating fever and convulsion, and to relieving blood bleeding, it is available to substitute SAH with GH or TAH.

### Conclusions

The overall proteomes of the different horns and traditional bio-effect evaluation were highly similar, and only the relative amounts of proteins in the soluble and insoluble fractions differed between samples, only the dose level and effect strength showed difference between samples. In the present study, it provides evidences on using GH, WBH, or YH as substitutes of RH and SAH in rat experimental models, it also suggests that RH and SAH are not especially unique or indispensable. Proteomic analysis combined with bio-effect evaluation therefore provide a strategy to search and evaluate sustainable animal horns alternatives as available substitutes of endangered animal horns derived TCMs.

## Materials and Methods

### Sample preparation

**Figure 10:**
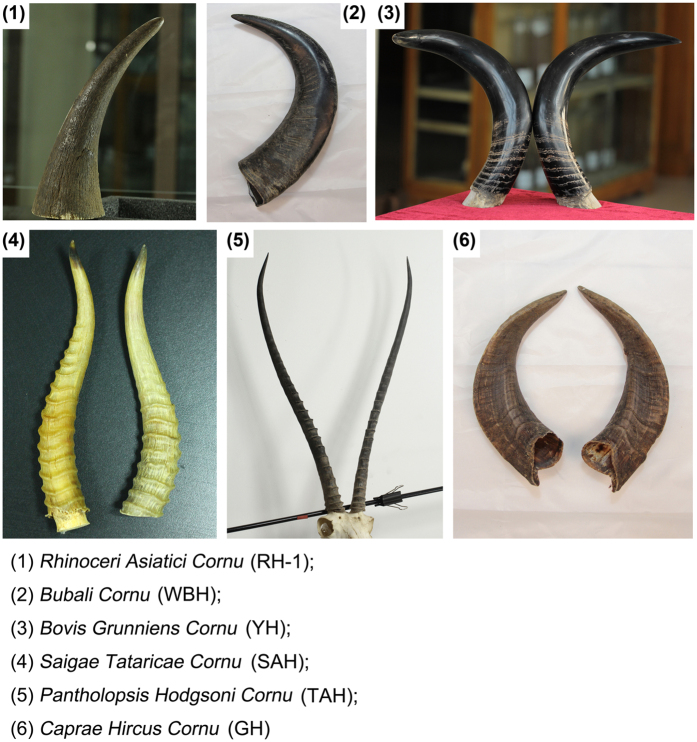
Pictures of animal horn samples.

*Rhinoceri Asiatici Cornu* (RH-1), *Rhinoceri Africani Cornu* (RH-2), and *Pantholopsis Hodgsoni Cornu* (Tibetan antelope horn, TAH) were purchased from Jiangsu Medicinal materials company (Permission State Forestry Administration of China for purchasing Rhino horn samples, 2003, No. 7). *Saigae Tataricae Cornu* (SAH), *Bubali Cornu* (WBH) and *Caprae Hircus Cornu* (GH) were purchased from Jiangsu Medicine Company (Jiangsu, China). *Bovis Grunniens Cornu* (YH) was purchased from a slaughter house located on Linkuo North Road in Lhasa, Tibet. All horn samples were authenticated by Prof. Dr. Jin-ao Duan. All horn products were obtained as pieces form initially, and then were powdered. Detailed information is listed in Table S33 and Fig. 10.

### Animals

Adult New Zealand rabbits (2.0–2.5 kg) and mice (18–20 g) of both sexes, obtained from the Center of Experimental Animals, China Pharmaceutical University. Animals were housed under standard conditions of temperature (22 ± 2 °C); relative humidity (55 ± 5%) and light (12 h light/dark cycles) were used. The animals were fed with standard diet and water *ad libitum*. Animal welfare and experimental procedures were strictly in accordance with the Guide for the Care and Use of Laboratory Animals. This study was approved by Nanjing University of Chinese Medicine.

### Antipyretic activity test

The antipyretic activity test was performed according to the previous method^25^. Antipyretic activity was determined in rabbits using six animals of both sexes for each group. Rabbits presenting an initial rectal temperature between 38.5–39 °C were selected for the antipyretic tests. Hyperthermia was induced by i.v. injection of *Escherichia coli* endotoxin at a dose of 20 ng/kg. Rectal temperature was measured 6 h after the endotoxin injection, and rabbits, developing significant hyperthermia were used only. Then the rabbits were orally administered with horn powder (dosage of horns was shown in Table S30), and their model group (saline), positive group (paracetamol at 20 mg/kg body weight) and rectal temperatures recorded at 15, 30, 45, 60, 90, 120, 180, and 240 min after drug treatment.

The antipyretic activity test on rat was also performed according to the previous method^6^. Antipyretic activity was determined in rats using eight animals of both sexes for each group. Rats with an initial rectal temperature of 37–38 °C were selected for the study. Hyperthermia was induced by subcutaneous injection of 20% (w/v) yeast (Anqi Co., Ltd. Hubei, China) in 0.9% sterile saline (10 mL/kg). Rectal temperatures were measured 6 h after the yeast injection and only rats developing significant hyperthermia were used selected. And then rats were treated orally with horn powder (dosage of horns was shown in Table S30), and their model group (saline), positive group (Aspirin at 100 mg/kg body weight) and rectal temperatures recorded at 60 and 120 min after drug treatment. Blood samples of 400 L were collected in heparin containing tubes from epicanthic veins of rats by capillary tube at 60 and 120 min after drug treatment, and centrifuged at 4000 rpm for 10 min. The plasma were collected and TNF-α, IL-6, and PGE_2_ levels were measured by enzyme linked immunosorbent assay (ELISA) kit (Yi Fei Xue biotech. Nanjing, China).

### Sedative activity test

The test was performed according to the method of Connor with slight modification^26^. This test measures exploration and voluntary locomotion within an enclosed area was used to evaluate the sedative activity of the horns. Objective values for spontaneous motor activity were obtained with a photoactometer. Mice were placed individually in a black chamber with a screen floor and a light-tight lid. Six beams of red light were focused 2 cm above the floor onto photocells on the opposite side. Each beam interruption registered as an event on an external counter. The floor of the chamber was wiped clean with 5% (v/v) alcohol before each use. The mice were oral administered with horn powder (dosage of horns was shown in Table S30), and their control (saline), positive group (estazolam at 0.5 mg/kg body weight). Mice were placed in the chamber 30 min after orally administration. They were allowed to acclimate for 2 min, and then light beam breaks were counted for the next 2 min.

### Procoagulant activity test

The blood clotting time (CT) experiment was performed according to the method of Singh to evaluate the procoagulant activity of horns, with slight modification^27^. Mice were divided into six groups of ten animals each. The mice were orally administered with horn powder (dosage of horns was shown in Table S30), and their control (saline), positive group (aminomethylbenzoic at 36.7 mg/kg body weight) once a day for 3 days. 1 h after last orally administration, blood samples were taken with the help of a glass capillary from orbital plexus of the eye of each mouse and the time was noted. Small pieces of capillary were broken from one end at every 15 s till fibrin threads of blood appeared between the broken ends of capillary.

### Quantification of therapeutic effects

To quantify the antipyretic and sedative activity of horns, area under curve (AUC) were introduced to evaluate the effect and effective time in pharmacology experiments. AUC was calculated by Graphpad Prism 5 software.

### Extraction and digestion of animal horns

It was performed as described with slight modifications^15^. Typically, 20 mg of animal horn powder was immersed in 4 ml of 2% sodium dodecyl sulfate (SDS), 50 mM sodium phosphate (pH 7.8), 20 mM DTT and incubated overnight at 65 °C. The soluble and insoluble materials were separated by centrifugation, and the insoluble material was resuspended in 2% SDS, 50 mM sodium phosphate (pH 7.8), 20 mM DTT, incubated overnight at 65 °C and extracted as before. After four such extractions, approximately 85% of the total protein was extracted.

Aliquots of the soluble (first extract) and insoluble material were incubated for 0.5 h in 2% SDS, 20 mM DTT, and 50 mM phosphate buffer (pH 7.8) then incubated at room temperature for an additional 0.5 h following addition of 40 mM iodoacetamide. Proteins were precipitated from the soluble extract by addition of 3 volumes of ethanol. The soluble and insoluble protein samples were rinsed with 70% ethanol, then with freshly prepared 0.1 M ammonium bicarbonate, and were finally resuspended in fresh 0.1 M ammonium bicarbonate adjusted to 2 M urea. To each suspension bovine L-1-tosylamido-2-phenylethyl chloromethylketone-treated trypsin (Worthington, Lakewood, Colorado, USA) was added to 1% (w/v). After 6–8 h at 37 °C, samples were stirred overnight at room temperature. Most of the detergent-insoluble material was solubilized, and virtually all of the detergent-soluble material was solubilized in this way.

### Mass spectrometry

Mass spectrometric experiments were performed using a nano LC LTQ-Orbitrap mass spectrometer (Thermo Fisher Scientific, Bremen, Germany) equipped with a nano-electrospray ion source and an Easy II nano LC, as described previously with some modifications^28^. Animal horn samples were divided into soluble and insoluble fractions as described above, and 5 μL samples (1 μg of total protein) were loaded onto a self-packed 5 μm Reprosil C18AQ column (75 μm × 150 mm). The mobile phase consisted of acetonitrile/formic acid/water (2/0.2/98, v/v/v) for buffer A and acetonitrile/formic acid/water (80/20/0.2, v/v/v) for buffer B. Processed samples were analyzed using a 150 min gradient from 2% to 30% B. The LTQ-Orbitrap was operated in data-dependent acquisition mode to automatically alternate between a full scan (m/z 300–2000) in the Orbitrap and CID MS/MS scans in the linear ion trap. The ten most intense peptide ions were isolated for fragmentation. Helium was used as collision gas for CID. The normalized collision energy was 35% and the activation time was 30 ms. Unless otherwise stated, three replicate measurements were made at each MS setting. Data acquisition was controlled by Xcalibur 2.0.7 and Tune 2.4 software (Thermo Fisher Scientific).

### Protein identification

Tandem mass spectra were extracted with Xcalibur version 2.0.7. All MS/MS samples were analyzed using X!Tandem (thegpm.org; version TORNADO 2010.01.01.4) which was set up to search the bovidae_uniprot-taxonomy_9895 database (downloaded on the 26^th^ April, 2014). Spectra were also searched against an equal number of decoy sequences to estimate the false discovery rate (FDR) as described previously^28^. The specified enzyme was trypsin and up to two missed cleavages were allowed. Oxidation of methionine (+15.9949) and acetylation of the protein N-terminus (+42.0106) were specified as variable modifications and carbamidomethylation of cysteine (+57.0215) was specified as a fixed modification. All other parameters were default settings, including a fragment ion tolerance of 0.5 Da and a maximum precursor ion tolerance of 6 ppm after recalibration. Identified peptides were filtered to curate a dataset with an FDR of less than 1% at both the peptide and protein levels. Protein identifications were accepted if the probability was over 90% (as assigned by the Protein Prophet algorithm) and included at least two identified peptides.

### Statistical analysis

Data from the solubilized and particulate fractions were analyzed separately. Hierarchical clustering and principal component analysis (PCA) were performed using SPSS 16.0 and Simca-P.

### Ontology analysis of identified proteins

GI numbers of identified proteins were matched to the UniProtKB database (www.uniprot.org) to obtain Gene Ontology Annotation (GO) using the molecular functions and cellular component categories.

## Additional Information

**How to cite this article**: Liu, R. *et al*. Available sustainable alternatives replace endangered animal horn based on their proteomic analysis and bio-effect evaluation. *Sci. Rep.* **6**, 36027; doi: 10.1038/srep36027 (2016).

**Publisher’s note:** Springer Nature remains neutral with regard to jurisdictional claims in published maps and institutional affiliations.

## Supplementary Material

Supplementary Information

Supplementary Table S1

Supplementary Table S2

Supplementary Table S3

Supplementary Table S4

Supplementary Table S5

Supplementary Table S6

Supplementary Table S7

Supplementary Table S8

Supplementary Table S9

Supplementary Table S10

Supplementary Table S11

Supplementary Table S12

Supplementary Table S13

Supplementary Table S14

Supplementary Table S15

Supplementary Table S16

Supplementary Table S17

Supplementary Table S18

Supplementary Table S19

Supplementary Table S20

Supplementary Table S21

Supplementary Table S22

Supplementary Table S23

Supplementary Table S24

Supplementary Table S25

Supplementary Table S26

Supplementary Table S27

Supplementary Table S28

Supplementary Table S29

## References

[b1] But P. P., Lung L. C. & Tam Y. K. Ethnopharmacology of rhinoceros horn. I: Antipyretic effects of rhinoceros horn and other animal horns. J. Ethnopharmacol. 30, 157–168 (1990). 2255207 10.1016/0378-8741(90)90005-e

[b2] But P. P., Tam Y. K. & Lung L. C. Ethnopharmacology of rhinoceros horn. II: Antipyretic effects of prescriptions containing rhinoceros horn or water buffalo horn. J. Ethnopharmacol. 33, 45–50 (1991). 1943172 10.1016/0378-8741(91)90159-b

[b3] Mikulíková K. . Study of saiga horn using high-performance liquid chromatography with mass spectrometry. Scientific World J. 2012, 759604 (2012). 10.1100/2012/759604 PMC3354689 22629195

[b4] Still J. Use of animal products in traditional Chinese medicine: environmental impact and health hazards. Complement Ther. Med. 11, 118–122 (2003). 12801499 10.1016/s0965-2299(03)00055-4

[b5] IUCN Red List Categories, IUCN Species Survival Commission, The World Conservation Union: Available at: http://www.iucnredlist.org (Accessed: March 2015).

[b6] Liu R., Wang M. & Duan J. A. Antipyretic and antioxidant activities of the aqueous extract of *Cornu Bubali* (water buffalo horn). Am. J. Chin. Med. 38, 293–306 (2010). 20387226 10.1142/S0192415X10007853

[b7] Luo J. Y. . Substitutes for endangered medicinal animal horns and shells exposed by antithrombotic and anticoagulation effects. J. Ethnopharmacol. 136, 210–216 (2011). 21549826 10.1016/j.jep.2011.04.053

[b8] Yan D. . Forensic DNA barcoding and bio-response studies of animal horn products used in traditional medicine. PLOS One 8, e55854 (2013). 23409064 10.1371/journal.pone.0055854 PMC3568084

[b9] Luo J. Y. . A strategy for trade monitoring and substitution of the organs of threatened animals. Sci. Rep. 3, 3108 (2013). 24173429 10.1038/srep03108 PMC3813934

[b10] Liu R. . Analysis of active components of rhinoceros, water buffalo and yak horns using twodimensional electrophoresis and ethnopharmacological evaluation. J Sep Sci 34, 354–362 (2011). 21268260 10.1002/jssc.201000617

[b11] Anonymous. Pharmacological experiments of Saiga antelope horn, goat horn, and sheep horn. Jiangsu Med 4, 57–59 (1976). [in Chinese].

[b12] Shen M. Q., Ye Q. Z., Ding Y. F. & Luo Y. H. Study on *Cornu Bos grunniens* from Tibet substituting Cornu Rhinoceri. J. Chin. Med. Mat. 31, 813–815 (2008). [in Chinese]. 18998561

[b13] Hieronymus T. L., Witmer L. M. & Ridgely R. C. Structure of white rhinoceros (*Ceratotherium simum*) horn investigated by X-ray computed tomography and histology with implications for growth and external form. J. Morphol. 267, 1172–1176 (2006). 16823809 10.1002/jmor.10465

[b14] Rice R. H., Xia Y., Alvarado R. J. & Phinney B. S. Proteomic analysis of human nail plate. J. Proteome Res. 9, 6752–6758 (2010). 20939611 10.1021/pr1009349 PMC3007598

[b15] Lee Y. J., Rice R. H. & Lee Y. M. Proteome analysis of human hair shaft: from protein identification to posttranslational modification. Mol. Cell Proteomics 5, 789–800 (2006). 16446289 10.1074/mcp.M500278-MCP200

[b16] Marshall R. C., Orwin D. F. G. & Gillespie J. M. Structure and biochemistry of mammalian hard keratin. Electron Microsc. Rev. 4, 47–83 (1991). 1714783 10.1016/0892-0354(91)90016-6

[b17] Rice R. H., Winters B. R., Durbin-Johnson B. P. & Rocke D. M. Chicken corneocyte cross-linked proteome. J. Proteome Res. 12, 771–776 (2013). 23256538 10.1021/pr301036k PMC3569041

[b18] Koehn H. . The proteome of the wool cuticle. J. Proteome Res. 9, 2920–2928 (2010). 20423113 10.1021/pr901106m

[b19] Folk J. E. & Finlayson J. S. The ε-(γ-glutamyl)lysine crosslink and the catalytic role of transglutaminases. Adv. Protein Chem. 31, 1–133 (1977). 73346 10.1016/s0065-3233(08)60217-x

[b20] Liu R., Wang M., Duan J. A., Guo J. M. & Tang Y. P. Purification and identification of three novel antioxidant peptides from *Cornu Bubali* (water buffalo horn). Peptides 31, 786–793 (2010). 20206218 10.1016/j.peptides.2010.02.016

[b21] Rice R. H., Wong V. J. & Pinkerton K. E. Ultrastructural visualization of cross-linked protein features in epidermal appendages. J. Cell Sci. 107, 1985–1992 (1994). 7983163 10.1242/jcs.107.7.1985

[b22] Editorial Committee of Administration Bureau of Traditional Chinese Medicine. Chinese Materia Medica (Zhonghua Bencao) vol. **27**, 734–738 (Shanghai Science & Technology Press, 2006). [in Chinese].

[b23] Wang F., Duan J. A., Qian D. W. & Li Y. B. Searching for substitutes for *Cornu Rhinoceri Asiatici* and *Cornu Saigae Tataricae* and evaluation (I). J. Nanjing TCM Univ. 23, 163–165 (2005). [in Chinese].

[b24] Wang F., Duan J. A., Qian D. W. & Li Y. B. Searching for substitutes for *Cornu Rhinoceri Asiatici* and *Cornu Saigae Tataricae* and evaluation (II). J. Nanjing TCM Univ. 23, 36–39 (2007). [in Chinese].

[b25] Backhouse C. N. . Active constituents isolated from Psoralea glandulosa L. with antiinflammatory and antipyretic activities. J Ethnopharmacol 78, 27–31 (2001). 11585684 10.1016/s0378-8741(01)00309-9

[b26] Connor J. D., Rostom A. & Makonnen E. Comparison of effects of khat extract and amphetamine on motor behaviors in mice. J Ethnopharmacol 81, 65–71 (2002). 12020929 10.1016/s0378-8741(02)00035-1

[b27] Singh S., Rehan H. M. & Majumdar D. K. Effect of Ocimum sanctum fixed oil on blood pressure, blood clotting time and pentobarbitone-induced sleeping time. J Ethnopharmacol 78, 139–143 (2001). 11694358 10.1016/s0378-8741(01)00336-1

[b28] Dietrich M. A. . Characterization of carp seminal plasma proteome in relation to blood plasma. J. Proteomics 98, 218–232 (2014). 24434589 10.1016/j.jprot.2014.01.005

