# Supplementary material for: Available sustainable alternatives replace endangered animal horn based on their proteomic analysis and bio-effect evaluation
Source: Sci Rep. 2016 Oct 27;6:36027. doi: 10.1038/srep36027 (PMC5082367; doi:10.1038/srep36027)
Supplement: Supplementary Information [file srep36027-s1.doc]

**Available sustainable alternatives replace endangered animal horn based on their proteomic analysis and bio-effect evaluation**

Rui Liu^1, 2, 3^, *, §,Fei Wang^4, 5^, §, Qiong Huang^6^, §, Jin-ao Duan^1, 3^, **, Pei Liu^1, 3^, Erxin Shang^1, 3^, Dong Zhu^2^, Hongmei Wen^1, 2^, Dawei Qian^1, 3^

*1. Jiangsu Collaborative Innovation Center of Chinese Medicinal Resources Industrialization, and National and Local Collaborative Engineering Center of Chinese Medicinal Resources Industrialization and Formulae Innovative Medicine, Nanjing 210023, PR China*

*2. Jiangsu Key Laboratory of Research and Development in Marine Bio-resource Pharmaceutics, Nanjing University of Chinese Medicine, Nanjing 210023, PR China*

*3. Jiangsu Key Laboratory for High Technology Research of TCM Formulae, Nanjing University of Chinese Medicine, Nanjing 210023, PR China*

*4. School of Pharmacy, Jiangsu University, Zhenjiang 212013, PR China*

*5. Suzhou Hospital of Traditional Chinese Medicine, Suzhou, 215009, PR China*

*6. The First Affiliated Hospital, Nanjing Medical University, Nanjing 210029, PR China*

§ The authors have contributed equally to this paper

**Captions of supplemental Tables**

Table S1, Keratins identified from all animal samples

Table S2, Cellular component analysis of RH-1 soluble sample

Table S3, Molecular function analysis of RH-1 soluble sample

Table S4, Cellular component analysis of RH-1 insoluble sample

Table S5, Molecular function analysis of RH-1 insoluble sample

Table S6, Cellular component analysis of RH-2 soluble sample

Table S7, Molecular function analysis of RH-2 soluble sample

Table S8, Cellular component analysis of RH-2 insoluble sample

Table S9, Molecular function analysis of RH-2 insoluble sample

Table S10, Cellular component analysis of SAH soluble sample

Table S11, Molecular function analysis of SAH soluble sample

Table S12, Cellular component analysis of SAH insoluble sample

Table S13, Molecular function analysis of SAH insoluble sample

Table S14, Cellular component analysis of TAH soluble sample

Table S15, Molecular function analysis of TAH soluble sample

Table S16, Cellular component analysis of TAH insoluble sample

Table S17, Molecular function analysis of TAH insoluble sample

Table S18, Cellular component analysis of WBH soluble sample

Table S19, Molecular function analysis of WBH soluble sample

Table S20, Cellular component analysis of WBH insoluble sample

Table S21, Molecular function analysis of WBH insoluble sample

Table S22, Cellular component analysis of YH soluble sample

Table S23, Molecular function analysis of YH soluble sample

Table S24, Cellular component analysis of YH insoluble sample

Table S25, Molecular function analysis of YH insoluble sample

Table S26, Cellular component analysis of GH soluble sample

Table S27, Molecular function analysis of GH soluble sample

Table S28, Cellular component analysis of GH insoluble sample

Table S29, Molecular function analysis of GH insoluble sample

Table S30, Dosage of pharmacological experiment

Table S31, The effect of animal horns on fever rabbits. Values were expressed as mean ± SD (n = 6)

Table S32, Effects of animal horns given on spontaneous motor activity in mice. Values were expressed as mean ± SD (n = 10)

Table S33, The effect of animal horns on fever rats. Values were expressed as mean ± SD (n = 8)

**Table S30**. Dosage of pharmacological experiment

| Sample | | Rabbit dosage (g/kg) | Rat dosage (g/kg) | Mouse dosage (g/kg) | |
| --- | --- | --- | --- | --- | --- |
|  |  | Antipyretic activity | | Sedative activity | Procoagulant activity |
| RH-1 | Low | 0.015 |  | 0.055 | 0.055 |
|  | Mid | 0.03 |  | 0.11 | 0.11 |
|  | High | 0.06 | 0.12 | 0.22 | 0.22 |
| RH-2 | Low | 0.015 |  | 0.055 | 0.055 |
|  | Mid | 0.03 |  | 0.11 | 0.11 |
|  | High | 0.06 | 0.12 | 0.22 | 0.22 |
| SAH | Low | 0.015 |  | 0.055 | 0.055 |
|  | Mid | 0.03 |  | 0.11 | 0.11 |
|  | High | 0.06 | 0.12 | 0.22 | 0.22 |
| TAH | Low | 0.1875 |  | 0.6875 | 0.6875 |
|  | Mid | 0.375 |  | 1.375 | 1.375 |
|  | High | 0.75 | 1.5 | 2.75 | 2.75 |
| WBH | Low | 0.375 |  | 1.375 | 1.375 |
|  | Mid | 0.75 |  | 2.75 | 2.75 |
|  | High | 1.5 | 3 | 5.5 | 5.5 |
| YH | Low | 0.375 |  | 1.375 | 1.375 |
|  | Mid | 0.75 |  | 2.75 | 2.75 |
|  | High | 1.5 | 3 | 5.5 | 5.5 |
| GH | Low | 0.1875 |  | 0.6875 | 0.6875 |
|  | Mid | 0.375 |  | 1.375 | 1.375 |
|  | High | 0.75 | 1.5 | 2.75 | 2.75 |

**Table S31**. The effect of animal horns on fever rabbits. Values were expressed as mean ± SD (n = 6)

|  | | 0 min | 15 min | 30 min | 45 min | 60 min | 90 min | 120 min | 180 min | 240 min | AUC_240 min_ |
| --- | --- | --- | --- | --- | --- | --- | --- | --- | --- | --- | --- |
| Control | | 0.00±0.15 | 0.08±0.07 | 0.13±0.11 | 0.14±0.12 | 0.06±0.09 | 0.15±0.22 | 0.09±0.12 | 0.11±0.12 | 0.12±0.13 | 233.8 |
| Model | | 0.65±0.22### | 0.85±0.39## | 0.96±0.33### | 1.15±0.24### | 1.04±0.27### | 1.05±0.33## | 0.98±0.31### | 0.99±0.29### | 0.87±0.44### | 25.4 |
| Positive control | | 0.65±0.08 | 0.92±0.19 | 0.94±0.11 | 0.85±0.33 | 0.61±0.31* | 0.56±0.17* | 1.03±0.54 | 0.90±0.52 | 0.89±0.46 | 203.1 |
| RH-1 | Low | 0.63±0.21 | 0.94±0.27 | 0.96±0.36 | 1.10±0.47 | 0.98±0.16 | 1.10±0.26 | 1.25±0.35 | 1.15±0.50 | 1.01±0.64 | 260.3 |
|  | Mid | 0.66±0.23 | 0.87±0.24 | 0.83±0.24 | 0.74±0.30* | 0.53±0.21* | 0.75±0.46 | 0.83±0.32 | 1.11±0.73 | 1.00±0.75 | 209.9 |
|  | High | 0.62±0.36 | 0.77±0.29 | 0.69±0.32 | 0.52±0.16** | 0.43±0.17** | 0.56±0.31* | 0.59±0.31 | 0.83±0.65 | 0.78±0.57 | 160.6 |
| RH-2 | Low | 0.71±0.31 | 0.82±0.28 | 1.05±0.21 | 1.06±0.28 | 0.98±0.26 | 0.83±0.12 | 0.68±0.22 | 0.79±0.50 | 0.84±0.46 | 199.4 |
|  | Mid | 0.61±0.18 | 0.74±0.21 | 0.91±0.23 | 0.74±0.09** | 0.95±0.43 | 0.73±0.30 | 1.06±0.56 | 1.11±0.70 | 1.06±0.71 | 229.8 |
|  | High | 0.64±0.20 | 0.98±0.46 | 0.83±0.16 | 0.48±0.23** | 0.50±0.19** | 0.54±0.23* | 0.78±0.44 | 0.99±0.48 | 0.95±0.58 | 189.6 |
| SAH | Low | 0.62±0.25 | 0.97±0.09 | 1.08±0.12 | 0.85±0.28 | 0.95±0.28 | 1.09±0.32 | 1.05±0.32 | 0.93±0.27 | 0.83±0.45 | 230.2 |
|  | Mid | 0.63±0.19 | 0.79±0.34 | 0.96±0.27 | 0.83±0.34 | 0.69±0.16* | 0.67±0.34 | 0.64±0.28 | 0.79±0.22 | 0.77±0.34 | 178.4 |
|  | High | 0.68±0.38 | 1.14±0.42 | 0.99±0.48 | 0.91±0.40 | 0.51±0.27* | 0.83±0.44 | 0.90±0.46 | 0.86±0.34 | 0.88±0.38 | 205.6 |
| TAH | Low | 0.68±0.15 | 0.85±0.20 | 1.04±0.29 | 0.95±0.26 | 1.09±0.16 | 0.98±0.28 | 0.98±0.31 | 1.32±0.20 | 1.20±0.55 | 260.9 |
|  | Mid | 0.64±0.21 | 0.72±0.27 | 0.82±0.28 | 0.90±0.20 | 0.83±0.15 | 0.96±0.09 | 0.96±0.27 | 1.00±0.42 | 1.01±0.37 | 222.4 |
|  | High | 0.66±0.27 | 0.96±0.41 | 1.10±0.23 | 0.88±0.19 | 0.88±0.30 | 0.62±0.24* | 0.98±0.29 | 1.05±0.39 | 1.00±0.57 | 224.6 |
| WBH | Low | 0.69±0.36 | 1.17±0.39 | 0.98±0.38 | 0.83±0.42 | 0.70±0.31 | 0.64±0.16* | 0.83±0.10 | 0.96±0.24 | 1.03±0.32 | 210.7 |
|  | Mid | 0.69±0.16 | 0.74±0.27 | 0.84±0.27 | 1.02±0.33 | 1.01±0.25 | 0.67±0.16* | 0.96±0.43 | 1.06±0.26 | 1.11±0.36 | 227.1 |
|  | High | 0.64±0.25 | 0.80±0.39 | 0.74±0.09 | 0.68±0.45 | 0.64±0.45 | 0.53±0.27* | 0.61±0.30 | 0.67±0.29 | 0.70±0.25 | 157.1 |
| YH | Low | 0.69±0.24 | 1.01±0.28 | 1.01±0.29 | 0.94±0.36 | 1.00±0.34 | 1.04±0.39 | 1.15±0.15 | 1.20±0.43 | 0.99±0.42 | 256.7 |
|  | Mid | 0.63±0.30 | 0.78±0.29 | 1.04±0.30 | 0.93±0.25 | 0.91±0.25 | 0.91±0.22 | 0.89±0.26 | 0.87±0.23 | 0.83±0.30 | 210.9 |
|  | High | 0.62±0.18 | 0.68±0.24 | 0.81±0.39 | 0.92±0.20 | 0.82±0.27 | 0.54±0.30* | 0.58±0.46 | 0.88±0.53 | 0.82±0.58 | 179.0 |
| GH | Low | 0.66±0.27 | 0.92±0.24 | 1.04±0.26 | 0.94±0.30 | 1.05±0.25 | 0.95±0.26 | 0.99±0.24 | 1.11±0.30 | 1.00±0.38 | 241.7 |
|  | Mid | 0.65±0.22 | 0.79±0.21 | 0.92±0.27 | 0.86±0.28 | 0.85±0.25 | 0.83±0.28 | 1.1±0.30 | 1.00±0.39 | 1.02±0.43 | 227.6 |
|  | High | 0.65±0.28 | 0.83±0.31 | 0.93±0.28 | 0.74±0.22 | 0.69±0.18* | 0.83±0.31 | 0.78±0.29 | 0.91±0.49 | 0.86±0.49 | 198.3 |

##*p* < 0.01, ###*p* < 0.001, compared to the Control group;

**p* < 0.05, ***p* < 0.01, compared to the Model group

**Table S32**. Effects of animal horns given on spontaneous motor activity in mice. Values were expressed as mean ± SD (n = 10)

|  | | 0 min | 20 min | 40 min | 60 min | 90 min | 120 min | AUC_120 min_ |
| --- | --- | --- | --- | --- | --- | --- | --- | --- |
| Control | | 58.2±21.6 | 46.7±18.0 | 50.8±25.6 | 43.0±22.0 | 40.9±22.7 | 42±21.7 | 5464 |
| Positive control | | 61.5±23.6 | 28.8±16.2* | 30.4±10.4* | 24.8±11.9* | 20.3±16.9* | 25.6±11.5* | 3412 |
| RH-1 | Low | 68.0±18.9 | 45.4±16.0 | 34.9±13.3 | 32.7±21.3 | 24.4±20.0 | 22.9±14.9* | 4179 |
|  | Mid | 57.3±23.1 | 47.3±19.3 | 33.9±10.7 | 25.8±12.4* | 19.1±9.6* | 28.4±28.5 | 3841 |
|  | High | 64.2±18.2 | 39.8±27.9 | 26.3±13.7* | 25.3±15.0* | 35.3±21.5 | 34.2±21.8 | 4168.5 |
| RH-2 | Low | 69.7±19.0 | 46.2±24.9 | 32.8±21.7 | 31.3±14.7 | 41.0±19.8 | 32.8±23.1 | 4781.5 |
|  | Mid | 67.9±24.0 | 32.3±13.1 | 25.2±13.1* | 21.4±10.3* | 33.7±12.9 | 40.2±18.8 | 3978 |
|  | High | 63.3±16.6 | 43.3±17.1 | 26.2±11.3* | 24.4±14.8* | 31.6±21.7 | 34.4±31.8 | 4097 |
| SAH | Low | 60.7±14.9 | 58.3±34.2 | 46.9±29.9 | 37.9±29.4 | 35.8±27.1 | 32.0±19.7 | 5212.5 |
|  | Mid | 68.4±32.4 | 59.6±25.4 | 54.7±27.1 | 30.0±16.9 | 30.3±22.4 | 39.4±22.5 | 5220 |
|  | High | 67.4±22.9 | 40.5±23.6 | 41.9±28.6 | 26.6±10.5* | 41.0±24.8 | 30.4±17.7 | 4673 |
| TAH | Low | 69.3±11.8 | 35.2±16.4 | 45.3±23.3 | 34.6±25.0 | 29.8±21.0 | 29.5±16.0 | 5309 |
|  | Mid | 63.9±15.7 | 42.9±13.7 | 41.2±19.4 | 33.4±19.0 | 34.6±23.4 | 33.7±32.0 | 4699.5 |
|  | High | 68.5±15.1 | 46.4±18.4 | 39.7±16.6 | 34.9±17.7 | 44.0±24.9 | 47.3±33.8 | 4504.5 |
| WBH | Low | 68.1±21.8 | 43.3±30.5 | 39.4±24.8 | 26.2±14.6 | 29.2±22.8 | 32.2±15.3 | 4349 |
|  | Mid | 67.3±12.6 | 48.3±28.2 | 35.0±23.6 | 24.6±19.1 | 28.3±14.0 | 23.6±12.3* | 4157 |
|  | High | 60.2±27.7 | 46.1±24.0 | 45.4±29.7 | 25.2±13.2* | 34.4±29.0 | 29.6±17.2 | 4538 |
| YH | Low | 62.6±22.6 | 53.1±19.3 | 43.6±22.7 | 34.2±22.9 | 33.6±20.5 | 38.2±20.2 | 4996 |
|  | Mid | 61.7±19.6 | 47.0±22.9 | 49.8±21.2 | 38.8±27.8 | 38.0±15.5 | 39.9±33.2 | 5261.5 |
|  | High | 56.2±16.0 | 48.3±19.8 | 30.1±16.1 | 23.5±16.1* | 28.7±13.8 | 30.8±18.3 | 4040.5 |
| GH | Low | 65.3±27.1 | 44.5±19.8 | 42.2±21.7 | 30.7±17.9 | 35.6±23.0 | 40.9±22.6 | 4836 |
|  | Mid | 66.8±15.4 | 43.3±16.3 | 45.2±24.2 | 31.1±20.6 | 32.7±22.5 | 38.9±15.9 | 4780 |
|  | High | 65.8±20.5 | 38.9±27.6 | 38.7±16.9 | 28.8±18.8* | 31.4±20.6 | 34.8±22.0 | 4394 |

**p* < 0.05, compared to the Control group

**Table S33**. The effect of animal horns on fever rats. Values were expressed as mean ± SD (n = 8)

|  | 0 min | 60 min | | | | 120 min | | | |
| --- | --- | --- | --- | --- | --- | --- | --- | --- | --- |
|  | ΔT (ºC) | ΔT (ºC) | TNF-α (ng/L) | IL-6 (ng/L) | PGE_2_ (ng/L) | ΔT (ºC) | TNF-α (ng/L) | IL-6 (ng/L) | PGE_2_ (ng/L) |
| Control | 0.15±0.17 | 0.45±0.13 | 40.9±13.3 | 23.5±6.7 | 62.0±1.8 | 0.18±0.22 | 57.5±3.5 | 33.3±3.7 | 61.3±2.4 |
| Model | 1.20±0.25### | 1.33±0.30## | 91.0±27.3## | 49.1±30.1# | 64.7±3.1# | 1.65±0.19### | 63.0±6.1# | 37.2±3.2# | 67.2±6.7# |
| Aspirin | 1.23±0.13 | 0.17±0.29** | 73.1±23.5 | 44.5±14.3 | 61.9±1.4* | -0.55±0.71*** | 48.1±10.9** | 29.2±4.6** | 53.0±7.9** |
| RH-1 | 1.15±0.33 | 0.90±0.54 | 51.0±10.8** | 23.3±8.0* | 60.6±1.6** | 0.70±0.23*** | 64.5±6.2 | 32.0±5.2* | 60.5±4.6* |
| RH-2 | 1.00±0.25 | 1.18±0.25 | 77.2±20.9 | 23.6±9.5* | 63.4±4.1 | 1.15±0.17** | 63.2±6.6 | 32.3±5.8 | 67.4±7.0 |
| SAH | 0.98±0.15 | 0.85±0.17* | 67.4±11.7* | 41.8±13.1 | 61.2±2.4* | 1.01±0.17** | 53.2±9.7* | 31.0±5.7* | 68.1±8.7 |
| TAH | 1.17±0.10 | 1.25±0.21 | 62.4±24.6* | 28.4±7.1 | 63.1±4.5 | 1.00±0.26* | 54.2±5.5** | 29.1±7.6* | 74.6±12.1 |
| WBH | 1.18±0.61 | 1.18±0.53 | 74.8±32.6 | 39.2±10.8 | 64.0±2.6 | 1.20±0.27* | 52.2±7.2** | 31.1±5.6* | 57.5±3.1** |
| YH | 1.00±0.29 | 1.40±0.29 | 58.1±36.8 | 36.3±14.5 | 62.9±1.2 | 1.43±0.31 | 49.3±10.8** | 31.7±5.7* | 72.4±17.7 |
| GH | 1.05±0.46 | 1.33±0.33 | 56.1±22.9* | 33.5±13.8 | 61.2±2.4* | 1.08±0.39* | 48.2±7.1** | 32.2±3.6* | 59.9±13.8 |

##*p* < 0.01, ###*p* < 0.001, compared to the Control group;

**p* < 0.05, ***p* < 0.01, ****p* < 0.001, compared to the Model group
